# Tiger density in a tropical lowland forest in the Eastern Himalayan Mountains

**DOI:** 10.1186/2193-1801-3-462

**Published:** 2014-08-24

**Authors:** Randeep Singh, Devendra Singh Chauhan, Sudhanshu Mishra, Paul R Krausman, Surendra Prakash Goyal

**Affiliations:** Wildlife Institute of India, Post Box # 18, Dehradun, Uttarakhand 248 001 India; Boone and Crockett Program in Wildlife Conservation, University of Montana, Missoula, MT 59812 USA

**Keywords:** Camera-trap, Northeast India, *Panthera tigris*, SECR, Semi-evergreen

## Abstract

Tropical evergreen forests in northeast India are a biological hot spot for conservation of flora and fauna. Little is known, however, about tiger abundance, which is a flagship species for tropical evergreen forests. Our objective was to document the capture rate and population density of tigers based on spatial explicit capture-recapture (SECR) approaches using camera trap data in an intensive study area (ISA) of 158 km^2^ in Pakke Tiger Reserve (PTR) during March to May 2006. The Reserve lies in the foothills of the Eastern Himalayan Mountains, northeast India. We monitored 38 camera traps in ISA for 70 days and documented 10 photo-captures of tigers (5 left and 5 right flanks) with an average trap success rate of 1.3 captures/100 trap days. The overall capture probability was 0.05. The tiger density estimated using a SECR model was 0.97 ± 0.23 individuals/100 km^2^. This is the first systematic sampling study in tropical semi evergreen forests of India, and information on capture rate and population density of tigers provides baseline data from which to determining changes in the future to assist conservation.

## Introduction

It is important to have information on distribution, abundance, and trends for making effective conservation and management planning and policies of large carnivores occurring naturally at low densities (Nowell and Jackson 1996). Among the large carnivores, tiger (*Panthera tigris*) is a flagship species in many of the eco-regions of Asia. Habitat loss, prey depletion, forest fragmentation, poaching, skin trade, and retaliatory killing are the interrelated impacts responsible for decline in tiger populations across its range (Dinerstein et al. 2007). It is crucial to monitor and assess abundance and status of such vulnerable species targeted by hunters, to identify problems, so that remedial steps can be initiated, otherwise local extinction of such species may occur (Barber-Meyer 2010), even in protected areas (Reddy 2008).

Tropical semi-evergreen rain forests in Southeast Asia are hot spots of biodiversity, and the eastern Himalayan region, especially northeast India, has been identified as one of the most biodiverse regions of the world (Myers et al. 2000). Tigers naturally occurs in low densities in tropical rainforests and are difficult to detect (Lynam et al. 2009). The utility of remotely triggered camera-traps offer possibilities with elusive species to generate photographic evidence to estimate the abundance and density in which individual identification is possible from the coat pattern (i.e., tiger *Panthera tigris*; Karanth and Nichols 1998; Ramesh et al. 2012; Singh et al. 2013a; 2013b; 2014a).

Presently very little is known regarding the abundance and population density of tigers from the tropical semi-evergreen forest of northeast India. Our objective was to provide, baseline information on the capture rate and population density of tigers in Pakke Tiger Reserve (PTR), Arunachal Pradesh, India. The PTR is one of four designated tiger reserves in northeast India, where illegal hunting and logging are serious threats for the conservation of the species.

## Material and methods

### Study site

**Figure 1:**
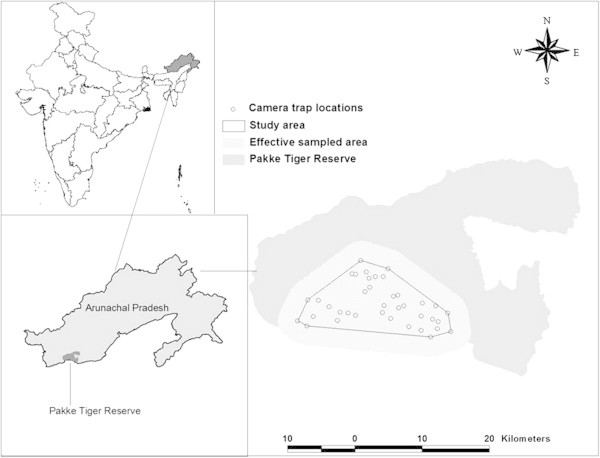
**Location of the intensive study area in Pakke Tiger Reserve in Arunachal Pradesh, India, with effectively sampled area and camera trap locations.**

Pakke Tiger Reserve (862 km^2^, 26°54′–27°16′N, and 92° 36′–93° 09′E) lies in the foothills of the Eastern Himalaya in the East Kameng District of Arunachal Pradesh (Figure 1) bordering Assam. It was declared a sanctuary in 1977, and has been recently declared a tiger reserve. The park is surrounded by contiguous forests on most sides and bounded by rivers in the east, west, and north. The terrain is undulating and hilly, with elevations from 150 to 2,000 m above sea level. At least 60 mammal species are reported from the park, including 7–8 species of felids, one bear, and two canid species, 16 viverrids, mustelids and herpestids, seven large herbivores, and four primate species (Datta and Goyal 1997). The vegetation of the park is classified as Assam valley tropical evergreen forest (Champion and Seth 1968). More than 20 villages and small settlements are located near the south-eastern boundary of the park adjacent to the Pakke River with an adult population of about 4,000 people (Datta and Goyal 1997). The area has great biological significance due to the richness of its flora and fauna, a result of its location in the Oriental and the Indo-Malayan realm, and has been considered as a hot spot for biodiversity (Myers et al. 2000).

### Sampling and analysis

Because of mountainous terrain and lack of an adequate road network, an intensive reconnaissance survey in dry stream beds, as suggested by Johnsingh et al. ( 2004), was conducted to record tracks and signs of large carnivores, especially tigers. Based on the distribution pattern of tracks, we identified an intensive study area (ISA) of 158 km^2^ in the PTR with minimal human disturbance. We selected 38 camera-trap locations based on the presence of tiger sign (i.e., tracks, scat, scrap marks). We conducted camera trapping, with active infrared camera-traps and13 TrailMaster TM 1550 plus camera kits (Goodson and Associates, Lenexa, Kansas, USA) between March and May 2006. Due to limited camera-traps, resource constraints, and lack of adequate roads for regular monitoring of cameras, we identified three trapping blocks (spatially separated) within the ISA and the cameras were deployed in a phased manner to systematically sample the area under "survey design 4" (Figure 1, Karanth and Nichols 2002). All camera-traps were operational for 24 hours. Each camera-trap location had single camera-traps positioned on either side of a trail. Combined captures from 1 day drawn from each block were used for each sampling occasion (Otis et al. 1978). To reduce the likelihood of tigers moving in and out of the trapping area undetected, we used a minimum trap spacing of 0.8 km and a maximum trap spacing of 2.2 km without any large holes in the sampling area (Karanth and Nichols 2002). We used the time and day imprinted in photo-capture to construct the capture matrix of individual tigers (Karanth and Nichols 2002). We tested the population closure assumption by using program CAPTURE (Rexstad and Burnham 1991). We constructed a capture history of tiger in spatial explicit capture-recapture (SECR) data format for analysis that considered a continuous 70-day sampling occasion (Singh et al. 2014b). We followed the SECR approach to obtain maximum likelihood density estimates for tigers using the camera trapping data (Efford 2011; Royle et al. 2009; Gopalaswamy et al. 2012).

We implemented the likelihood SECR models in program DENSITY 5.0 (Efford 2008; http://www.otago.ac.nz/density). We modeled the detection probability of each individual using the spatial detection function (Efford 2004), which was explained by two parameters (one-night detection probability at the center of an individual’s home-range, [g_o_] and a function of the scale of animal movements [σ]; Efford 2004). We used a half-normal detection function because it is appropriate for mark-recapture data from large carnivores. We evaluated the log likelihood function by integrating the Poisson distribution of the home range centers by adding a buffer of 10,000 m around the trapping grids (this distance was chosen to ensure that no individual outside of the buffered regions had any probability of being photographed by the camera trap during the survey; Zimmermann et al. 2013). The mean maximum distance moved was calculated using 1 recapture only. During our study one tiger recapture approximates a distance of 11 km, thus we used this distance to compute MMDM. For comparison we estimated tiger density using half the mean maximum distance moved (½ MMDM) approach (Karanth and Nichols 1998).

## Results

During the sampling period a sampling effort of 718 trap days over 158 km^2^, documented 10 tiger photographs (5 left flank photographs belonging to 4 individuals and 5 right flank photographs of 3 individuals) with a capture rate of 1.3 captures/100 trap days or 1 tiger every 71.8 trap-nights. Because there were more photo-captures of left flanks we used those data for density estimates. The statistical test for population closure in CAPTURE (Rexstad and Burnham 1991) supported the assumption that the sampled population was closed for the study period (*z* = 51.339, *P* = 0.09027). Using the M_h_ jackknife estimator the capture probability (p-ht) was estimated as 0.05. The maximum distance moved was 0.97 – 11.88 km and the ½ MMDM was 2.96 km. The boundary buffer width (W) was 2.96 km and the effectively sampled area (W) was 347 km^2^, thus the tiger estimated density (D [S.E]) was 1.15 ± 0.80 adult tigers/ 100 km^2^. The maximum likelihood (ML) tiger density was estimated as 0.97 ± 0.23 individuals/100 km^2^. The detection probability at the home range center (g_0_) was estimated at 0.0009 ± 0.0001. The sigma (a function of movement) value was 3,253 m ± 462 m.

## Discussion

Through, tropical semi-evergreen dense forests of Southeast Asia are well known as hot spots in biodiversity, they are considered to be poor habitat for prey, and thus vary in their density of carnivores (Datta et al. 2008). In protected areas of tropical rain forests of northeast India, carnivores are rare (Karanth and Nichols 2000; Datta et al. 2008). At Namdapha Tiger Reserve in tropical ever green forest, northeastern India, camera traps failed to detect any photo-captures of tigers after 451 (Karanth and Nichols 2000) and 1,537 trap days (Datta et al. 2008), even though tiger’s were known to be present. Similar results were obtained in Protected Areas (PA) in northern and central Thailand; only a single tiger was detected in each survey (Lynam et al. 2001, 2006). Thus, most of the studies so far undertaken in tropical rainforests of Southeast Asia have documented low rates of capture (0.03 to 2.7) of tigers in comparison to other areas (Table 1). During our study we recorded 10 photo-captures of tigers after 70 days of sampling (1.3 capture/100 trap days). Similar in northern Myanmar, after 190 day of sampling only 12 captures of 6 tigers were recorded in 3 different protected areas (Lynam et al. 2009). In PTR, tigers were detected with very low encounter rates in camera-traps but they were captured evenly in all trapping areas, which indicate the species’ presence throughout PTR in low densities.

**Table 1:** **Comparison of tiger captures rate and density derived from camera traps in Pakke Tiger Reserve, India and other tropical rain forest in Southeast Asia**

Location	Code	Country	Total camera days	Total no. photos	Total no. tigers	Effective sampled area (km ^2^)	Tiger density (no./100 km ^2^)	Capture/100 trap days
Pakke Tiger Reserve (Present study)	PTR	India	748	10	4	347	1.15	1.3
Namdapha Tiger Reserve (Datta et al. 2008)	NTR	India	1537	0	0	1200	0	0
Gunung Leuser^a^	GL	Indonesia	2686	45	10	550	1.82	1.7
Bukit Barisan Selatan^a^	BBS	Indonesia	4064	19	9	836	1.08	0.5
Kerinchi Seblat^a^	KS	Indonesia	5316	62	16	800	2.00	1.2
Halabala WS, Narathiwa Province^a^	HWS	Thailand	999	9	2	166.7	1.20	0.9
Queen Sirikit Reserve Forest, Yala Province^a^	QSR	Thailand	683	17	3	166.7	1.80	2.5
Phu Khieo WS, Chaiyaphum Province^a^	PKWS	Thailand	989	3	1	86.2	1.16	0.3
Khao Yai NP, Nakhon Ratchasima Province^a^	KYNP	Thailand	647	2	1	83.3	1.20	0.3
Temenggor Forest Reserve, Perak^a^	TFR	Malaysia	812	8	2	86.2	0.32	1.0
Bintang Hijau Forest, Perak^a^	BHF	Malaysia	776	7	2	202	0.99	1.0
Gunung Tebu Forest Reserve, Terngganu^a^	GTF	Malaysia	807	12	1	188.7	0.53	1.5
Ulu Temaing Forest Reserve, Kelantan^a^	UTF	Malaysia	563	15	2	210.5	0.95	2.7
Taman Negara^a^	TN	Malaysia	1829	6	4	338.2	1.18	0.3
Bungo primary selectively logged forest^b^	BP	Sumatra	2750	63	10	441	2.95	2.3
Ipuh primary selectively logged forest^b^	IP	Sumatra	3255	64	15	1227	1.55	2.0
Gunung Basor Forest Reserve^a^	GBFR	Malaysia	2496	18	6	308	2.59	0.7

^a^Carbone et al. 2001.

^b^Linkie et al. 2008.

**Figure 2:**
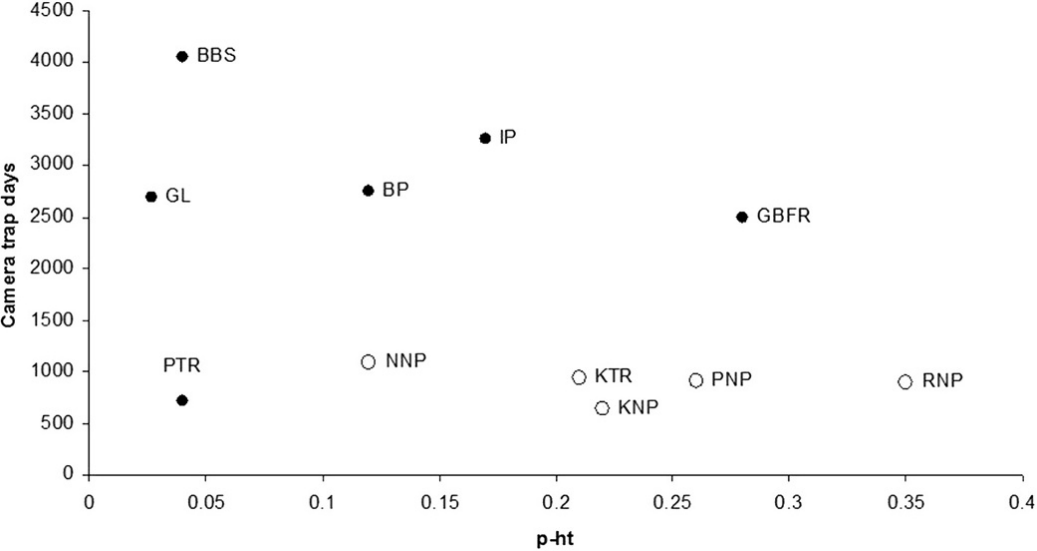
**Variation in capture probability (p-ht) in tigers of tropical semi-evergreen forest (dark circle) BBS = Bukit Barisan Selatan, Indonesia; GL = Gunung Leuser, Indonesia; PTR = Pakke Tiger Reserve, India; BP = Bungo Primary selectively logged forest, Sumatra; IP = Ipuh Primary selectively logged forest, Sumatra; GBFR = Gunung Basor Forest Reserve, Malaysia (Source; Carbone et al.** 2001 ) and Peninsular India (open circles) NNP = Nagarhole National Park, India; KTR = Kanha Tiger Reserve, India; KNP = Kaziranga National Park, India; *PNP = Panna National Park, India; RNP = Ranthambhore National Park, India (Source; Karanth and Nichols 1998 ; *Karanth et al. 2004 **b).**

The capture probability estimate in our study area is low (0.05), but also higher than minimal capture probability (0.03) required achieving reliable population density estimate (Harmsen, 2006). In a few areas of tropical semi-evergreen forests such as Gunung Leuser (GL) and Bukit Barisan Selatan, (BBS) of Indonesia, the capture probability (p-ht) remained very low (0.05) in spite of the very high number of camera trap days (2,686 to 4,064 trap days, respectively) (Figure 2). Thus, low capture probability should be expected in tropical rain forests because of low population densities of tiger.

Our results showed that the densities estimated under spatial (0.97 ± 0.23) and non-spatial approach (½MMDM; 1.15 ± 0.80) were almost similar. The results of density estimates (0.97 ± 0.23 adult tigers/100 km^2^) in PTR supports the fact that tigers occurs at low densities in tropical rain forests as reported in other rainforests (Table 1).

The reported population densities of tiger in tropical rain forest has been reported to range from 0.21– 2.95 tiger/100 km^2^, while in tropical dry and moist deciduous forest and grassland habitats the population densities of tiger were reported from 4 to 16 individuals per 100 km^2^ (Carbone et al. 2001; Karanth et al. 2004; Jhala et al. 2011). The variation in results between sites may be because of differences in vegetation, prey availability, and hunting pressure (Chapron et al. 2008). The tropical rainforests offer little primary productivity at ground level, and thus, the mammalian biomass is dominated by arboreal herbivores (Eisenberg 1980). A high proportion of the primary productivity in rain forests is in the canopy and is available to relatively small mammals, so food availability for large ungulates in tropical forests is low, hence low density of ungulates is expected (Glanz 1982). Because tiger abundance is related to prey abundance (Sunquist et al. 1999; Karanth et al. 2004), a lower tiger population density is likely in tropical rain forests.

Enforcement of India’s laws that entirely prohibit hunting of all wildlife is a challenge, especially in northeast India, where local tribes have a strong tradition of hunting. Although hunting has ritual, recreational, and subsistence value (Datta 2002; Hilaluddin et al. 2005; Mishra et al. 2006), it is also increasingly being driven by the high market value for derivatives from species such as tigers. Given the selective logging and hunting reported in tropical rain forests, the low density of species of carnivore including tigers may be adversely affected, leading to local extinction. As tigers have large habitat requirements, the effects of selective logging leading to the fragmentation and isolation of forest reserves, will severely affect the long-term viability of tiger populations across this landscape. Intensive and extensive monitoring of such elusive species that occur at a very low density and with a very low capture probability, require more effort in terms of money and time for monitoring purposes in tropical semi-evergreen forests. Therefore, we suggest a need for regular intensive and extensive monitoring of tiger (i.e., distribution, abundance, population density) and habitat characteristics, which may be undertaken in small forest blocks (100–150 km^2^) due to lack of easy accessibility of areas to avoid any local extinction in the future. This may be done by using camera trapping with increased capture days or widely used non-invasive genetic sampling (NGS) in carnivores (Mondol et al. 2009). It has also been suggested NGS is a suitable approach in areas, where large carnivores exhibited at low densities (Mondol et al. 2009), and enormous efforts would be needed to achieve reliable estimates (Foster and Harmsen 2012). Our results also demonstrate the need for further research on tiger ecology in tropical rain forests to inform decision makers and conservation planners of the conservation value of such habitats. Because this is one of the first systematic sampling studies in tropical semi evergreen forest of India, our information forms a base for detecting changes in populations in the future. We hope that future research will highlight the role of rain forests for tiger conservation and aid in providing effective tiger management guidelines for sustainable forest management in tropical rain forests.
